# Individualisierte Immuntherapie von Tumorerkrankungen mittels Peptidimpfstoffen – Funktioniert das vielleicht doch?

**DOI:** 10.1007/s00103-020-03227-3

**Published:** 2020-10-09

**Authors:** Hans-Georg Rammensee, Markus W. Löffler

**Affiliations:** 1 Interfakultäres Institut für Zellbiologie, Abteilung Immunologie, Eberhard Karls Universität Tübingen, Auf der Morgenstelle 15, 72076 Tübingen, Deutschland; 2 Deutsches Konsortium für Translationale Krebsforschung (DKTK) am Deutschen Krebsforschungszentrum (DKFZ), Partnerstandort Tübingen, Tübingen, Deutschland; 3 Exzellenzcluster iFIT (EXC 2180) „Individualisierung von Tumortherapien durch molekulare Bildgebung und funktionelle Identifizierung therapeutischer Zielstrukturen“, Tübingen, Deutschland; 4 Exzellenzcluster CMFI (EXC 2124) „Kontrolle von Mikroorganismen zur Bekämpfung von Infektionen“, Tübingen, Deutschland; 5 Klinik für Allgemeine, Viszeral- und Transplantationschirurgie, Universitätsklinikum Tübingen, Tübingen, Deutschland; 6 Abteilung Klinische Pharmakologie, Universitätsklinikum Tübingen, Tübingen, Deutschland

**Keywords:** Therapeutische Vakzinierung, Krebsantigene, Peptide, HLA-Moleküle, T‑Lymphozyten, Therapeutic vaccination, Cancer antigens, Peptides, HLA molecules, T lymphocytes

## Abstract

Bereits der Arzt und Forscher Paul Ehrlich stellte die These auf, dass das Immunsystem nicht nur Infektionen bekämpft, sondern auch gegen Krebs vorgehen kann. Über die möglichen positiven Auswirkungen einer simultanen Infektion auf den Verlauf einer Krebserkrankung wurde bereits im alten Ägypten ca. 2600 v. Chr. berichtet. Jedoch wurde erst ab den 1960er-Jahren klar, dass das Immunsystem Krebszellen gezielt bekämpfen kann, und erst ab den 1990er-Jahren wurde langsam aufgeklärt, wie dies vor sich geht.

Vor diesem Hintergrund sollen deshalb die Bemühungen der letzten 30 Jahre hinsichtlich der Entwicklung therapeutischer Impfungen gegen Krebserkrankungen kurz zusammengefasst und deren bisherige Erfolglosigkeit beleuchtet werden. Außerdem werden in einem Ausblick zukünftige eventuell Erfolg versprechende Entwicklungen in diesem Kontext diskutiert. Dabei werden die verfügbare wissenschaftliche Literatur, aber auch eigene Ergebnisse berücksichtigt.

Es ergeben sich ganz zentrale Fragen, etwa: Wie unterscheiden sich Krebszellen von normalen Zellen? Wie kann das Immunsystem diese Unterschiede erkennen? Was sind tumorspezifische Antigene? Warum müssen tumorspezifische Antigene in individueller Weise ausgesucht und angewendet werden? Wie induziert man eine effiziente Immunantwort? Welche pharmazeutischen Formulierungen, Adjuvanzien und Impfrouten sind effektiv?

Letztlich stellen wir dar, warum es sich möglicherweise doch lohnt, die bisher völlig erfolglose Peptidimpfung (gemessen an bisher zugelassenen Therapeutika) weiterzuverfolgen.

## Abstract (en)

The physician and scientist Paul Ehrlich put forward the thesis that the immune system not only fights infections but can also fight cancer. The possible positive effects of a simultaneous infection on the course of cancer were reported in ancient Egypt around 2600 BC. However, it was not until the 1960s that it became apparent that the immune system could specifically fight cancer cells, and it was not until the 1990s that researchers slowly clarified how this happens.

Against this background, the efforts over the last 30 years to develop therapeutic vaccines against cancers are briefly summarized, and their lack of success to date is highlighted. In addition, potentially promising future developments in this context are discussed. The available scientific literature as well as our own results are taken into account.

Central questions arise, such as the following: How do cancer cells differ from normal cells? How can the immune system recognize these differences? What are tumor-specific antigens? Why do they need to be selected and applied in an individualized fashion? How can an efficient immune response be induced? Which pharmaceutical formulations, adjuvants, and vaccination routes are effective?

Finally, we explain why it may still be worth pursuing peptide vaccination, which has so far been completely unsuccessful (when measured in terms of already approved therapeutics).

## Einleitung und historische Hintergründe

Bereits der Arzt und Forscher Paul Ehrlich stellte die These auf, dass das Immunsystem nicht nur Infektionen bekämpft, sondern möglicherweise auch gegen Krebs vorgehen kann [1]. Über mögliche positive Auswirkungen einer Infektion auf den Verlauf einer Krebserkrankung wurde bereits lange davor berichtet, so durch den altägyptischen Gelehrten Imhotep (~2600 v. Chr.; [2]). Auch der Bonner Chirurg Wilhelm Busch beschrieb bereits 1867 den Versuch, durch gezieltes Herbeiführen einer Infektion eine Tumorpatientin zu heilen. Dabei legte er die krebskranke Frau in das leere Bett eines Patienten, der an Wundrose erkrankt war. Kurze Zeit später schrumpfte der lebensbedrohliche Tumor im Hals der jungen Frau, sie verstarb allerdings kurz darauf trotzdem [3]. Weitere richtungsweisende Versuche wurden durch den New Yorker Chirurgen William Coley 1891 veröffentlicht [4], der vermutlich indirekt, in Publikationen von Robert Koch, Louis Pasteur und Emil von Behring, von den Bonner Ergebnissen erfahren hatte. Dabei beschrieb er unter anderem die Behandlung von inoperablen Knochensarkomen durch Injektion von Bakterien oder bakteriellen Produkten, die später auch als Coley-Toxine bekannt werden sollten [5]. Insgesamt behandelte er so am Memorial Hospital in New York über tausend Patienten während seines Berufslebens.

Es dauerte allerdings noch bis in die 1960er-Jahre, bis erstmals klar gezeigt werden konnte, dass das Immunsystem in der Lage ist, Krebserkrankungen zu bekämpfen [6], wofür insbesondere die Pionierarbeit von George und Eva Klein wesentlich war [7]. Erst in den 1990er-Jahren wurde langsam klar, wie das genau funktioniert: Es sind vor allem die T‑Zellen, die durch Krebs verursachte Veränderungen in Zellen erkennen können. Dies basiert, wie immer bei T‑Zellen, auf der Erkennung von Peptiden auf dem Haupthistokompatibilitätskomplex (MHC für „major histocompatibility complex“). Diese beim Menschen als HLA („human leukocyte antigen“) bezeichneten Moleküle finden sich allgemein auf der Oberfläche von Körperzellen.

Sowohl die HLA-Moleküle als auch die entsprechenden Tumorantigene sind allerdings in jedem Menschen individuell bzw. in jedem Patienten anders. Daher muss eine effektive Krebsimmuntherapie praktisch immer eigens zugeschnitten werden. Tumorspezifische Peptide bzw. Tumorantigene wurden in den letzten 30 Jahren in einer Vielzahl von Verabreichungsformen zu experimentellen Krebsimmuntherapien verarbeitet und in einzelnen Fällen sowie in kleineren Studien durchaus erfolgreich eingesetzt. Die genannten Verabreichungsformen reichen von völlig undefinierten Tumorzelllysaten über einzelne oder mehrere definierte Antigene, wie sie in Zellen exprimiert werden, insbesondere in dendritischen Zellen, über Virus- oder Bakterienkonstrukte, Proteine und Nukleinsäuren (Desoxyribonukleinsäure (DNS) und Ribonukleinsäure (RNS)) bis hin zu den so codierten Peptiden selbst, die den eigentlichen Naturstoffen, so wie sie tatsächlich auf den Krebszellen selbst vorkommen, entsprechen.

In einem einzigen Fall führte bisher eine formal erfolgreich durchgeführte klinische Phase-III-Studie zur Zulassung eines therapeutischen Impfstoffs [8]. Dieser Impfstoff zur Behandlung des Prostatakarzinoms mit der Bezeichnung Sipuleucel‑T erhielt 2010 die Zulassung durch die US-amerikanische Arzneimittelbehörde FDA (Food and Drug Administration; [9]), in Europa hingegen war er nur für kurze Zeit zugelassen (September 2013 bis Mai 2015), bevor die Zulassung auf Antrag des Zulassungsinhabers widerrufen wurde [10]. Dabei ist zu vermuten, dass der Impfstoff wegen relativer Ineffektivität und sehr hoher Kosten wieder vom Markt genommen worden ist.

Von den zahllosen möglichen Verabreichungsformen dieser Krebsimmuntherapien erscheinen uns 2 besonders relevant und potenziell zielführend:

1. die im Hauptteil dieses Übersichtsartikels aufgeführte individualisierte Anwendung von tumorspezifischen Peptiden in synthetischer Form,
2. die Anwendung von individualisierten Boten-RNS-Impfstoffen, wie im Abschnitt „Individualisierte Vakzine“ aufgeführt.

Die gezielte Vakzinierung mit antigencodierender Boten-RNS (englisch „messenger RNA“ (mRNA)) wurde übrigens von der Abteilung Immunologie und dem Institut für Organische Chemie in Tübingen bereits 1998/1999 entwickelt und im Jahre 2000 erstmalig publiziert [11]. Im Jahre 2008 wurden dann die Ergebnisse einer ersten klinischen Studie veröffentlicht [12].

## Immunologische Erkennung von malignen Tumoren

### Wie unterscheiden sich Krebszellen von normalen Zellen?

**Figure 1:**
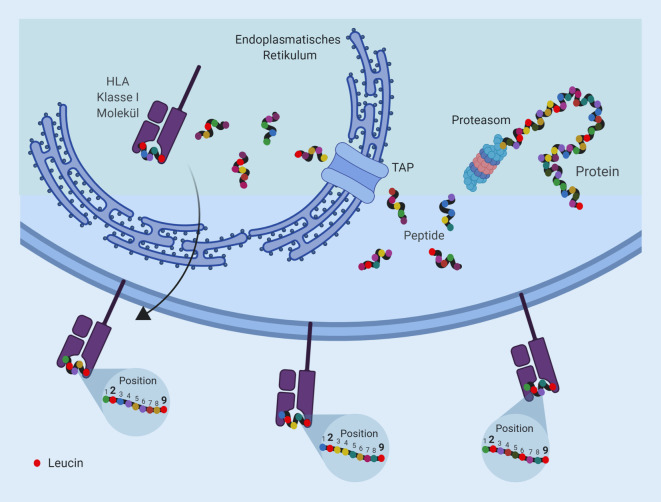

Fast jede Körperzelle hat HLA-Moleküle auf ihrer Zelloberfläche. Die physiologische Funktion dieser Strukturen ist es, Bruchstücke (Peptide genannt), die aus zellulären Eiweißen (Proteinen) entstammen, außen auf der Zelloberfläche zu zeigen und diese so T‑Zellen zugänglich zu machen (Abb. 1). Dadurch werden auch Veränderungen im Bestand dieser Peptide sichtbar und können so von T‑Zellen durchgemustert und erkannt werden. Diese Umstände führen dann dazu, dass etwa im Rahmen von Virusinfektionen oder bei malignen Erkrankungen neue dem Immunsystem bisher unbekannte Peptide gezeigt werden und dies dann zur Aktivierung virus- oder tumorspezifischer T‑Zellen führen kann, wodurch die infizierte bzw. veränderte Körperzelle dann abgetötet wird (Immunkontrolle).

### Wie kann das Immunsystem solche Unterschiede in Krebszellen erkennen und was sind tumorspezifische Antigene?

**Figure 2:**
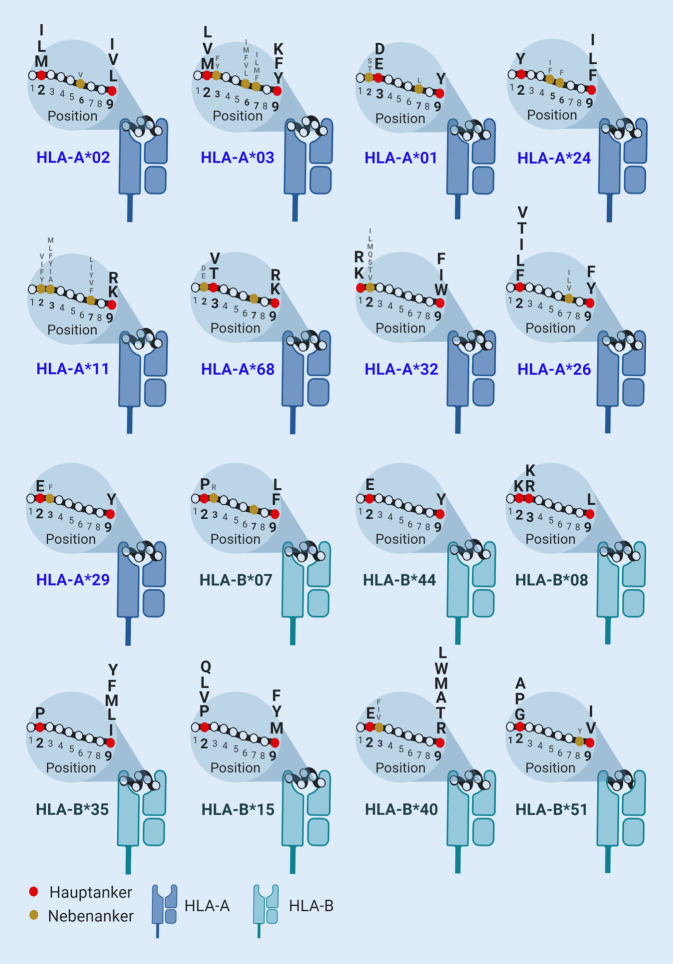

Unter normalen Umständen präsentiert jede Körperzelle viele Tausend unterschiedliche Peptide, die wiederum aus Tausenden von Proteinen der jeweiligen Zelle entstammen. Solche Peptide werden dann auf ihren HLA-Klasse-I-Molekülen präsentiert (Abb. 1). Während des normalen Stoffwechsels der Zelle entstehen diese kurzen Peptide aus Proteinen vor allem im Proteasom, einem zellulären Proteinkomplex wo Proteine gespalten und dann fragmentiert wieder freigesetzt werden. Obwohl die meisten dieser Peptide vollständig abgebaut werden, können einige der Peptide, die zufällig als Liganden in die Bindungsfurche von HLA-Molekülen passen, auf die Oberfläche der Zelle transportiert und dort präsentiert werden (Abb. 2). Im Gegensatz dazu werden auf HLA-Klasse-II-Molekülen längere Peptide präsentiert, die auch aus von der Zelle aufgenommenen externen Proteinen abstammen können, außerdem lassen sie sich auch nur auf der Zelloberfläche von wenigen spezialisierten Zelltypen bzw. nach Zytokininduktion finden. Nur in Verbindung mit dem HLA-Molekül können Peptide von T‑Zellen erkannt werden (was als MHC-Restriktion bezeichnet wird). Physiologisch entstehende Peptide, also Proteinfragmente aus normalen zellulären Proteinen, werden von T‑Zellen in der Regel nicht erkannt, da diese T‑Zellen im Thymus bereits während der negativen Selektion entfernt wurden (Selbsttoleranz). Wird eine Zelle aber etwa durch Viren infiziert, finden sich unter den vielen Tausend verschiedenen Peptiden auf der Zelloberfläche auch solche mit viralen Sequenzen, die dann als nicht körpereigen identifiziert und zur Aktivierung virusspezifischer T‑Zellen führen können. Das beschriebene Szenario trifft im Prinzip auch für virusinduzierte Krebsentitäten zu, wobei allerdings die meisten Krebsarten nicht oder zumindest nicht bekanntermaßen virusbedingt sind. Trotzdem gibt es vermutlich bei allen Krebsarten tumorspezifische HLA-präsentierte Peptide, obwohl diese normalerweise unbekannt bleiben. Ein bekanntes Beispiel für solche tumorspezifischen Peptide sind sogenannte mutierte Neoantigene, womit allgemein Peptide gemeint sind, die somatische Mutationen im Krebsgenom repräsentieren.

Solche Peptide weisen eine veränderte Aminosäuresequenz auf, verglichen mit allen entsprechenden normalen Proteinen, und sie können folglich, weil sie nicht einer körpereigenen Aminosäuresequenz entsprechen, von T‑Zellen erkannt werden. Viele Hinweise sprechen dafür, dass dies auch bei den durch Immuncheckpoint-Inhibitoren vermittelten T‑Zellantworten eine zentrale Rolle spielt und das Ausmaß solcher Veränderungen deshalb mit deren klinischer Wirksamkeit korreliert [13]. Zudem finden sich häufig Peptide auf Tumorzellen, die zwar nicht mutiert sind, aber auf normalen Körperzellen nicht vorkommen [14, 15]. Die Entstehung solcher tumorspezifischen Peptide und ihre Präsentation an T‑Zellen kann viele verschiedene Ursachen haben [16], zumeist sind diese aber noch nicht hinreichend erforscht und deshalb noch unbekannt. Trotzdem erscheinen solche häufig vorkommenden nichtmutierten Peptide, genauso wie die mutierten HLA-präsentierten Peptide, von denen es in der Regel nur sehr wenige gibt, für die Entwicklung von therapeutischen Vakzinen geeignet.

## Therapeutische Impfstoffe gegen Krebserkrankungen

### Warum müssen solche Impfstoffe individualisiert werden?

Das HLA-System ist üblicherweise für seine Rolle im Rahmen von Transplantationen bekannt; insbesondere bei der allogenen hämatopoetischen Stammzelltransplantation müssen die HLA-Eigenschaften von Spender und Transplantatempfänger zueinander passen. Die eigentliche Funktion des HLA ist aber die Antigenpräsentation (Abb. 1). So erkennen etwa CD8^+^-T-Zellen nur solche Peptide, wie sie auf den HLA-Klasse-I-Molekülen fast aller Körperzellen vorkommen. Die CD4^+^-T-Zellen erkennen dagegen nur Peptide auf HLA-Klasse-II-Molekülen, die normalerweise lediglich auf wenigen bestimmten Zelltypen vorkommen. Die HLA-codierenden Gene für die Klasse I umfassen HLA‑A, HLA‑B und HLA‑C und sind überaus polymorph; sie enthalten normalerweise über die Hälfte aller 4–5 Mio. der in der DNS codierten Einzelnukleotidpolymorphismen eines jeden Individuums [17]. Allein für die 3 HLA-Klasse-I-Loci (HLA‑A, HLA‑B und HLA-C) sind bisher knapp 17.000 verschiedene Allele beschrieben [18], zumeist werden davon pro Lokus beide Allele in Körperzellen exprimiert. Im besonderen Fall, wenn sich beide Allele entsprechen (Homozygotie), findet sich selbstverständlich nur ein einziges. Dadurch ergibt sich eine Vielfalt von allelspezifischen „Motiven“, weil verschiedene HLA-Moleküle durch ihre jeweiligen Bindungseigenschaften nur bestimmte Aminosäuren (Peptidspezifitäten) an definierten Stellen im präsentierten Peptid favorisieren (Abb. 2 illustriert die Motive einiger häufig vorkommender Allomorphe).

So weisen etwa HLA-A*02-gebundene Peptide die Aminosäuren Leucin (codiert als L) oder Isoleucin (I) sehr häufig an den Positionen 2 und 9 der Peptidsequenz auf. HLA-A*11-Moleküle favorisieren dagegen andere Aminosäuren, wie Lysin (K) oder Arginin (R), an Position 9 der Peptidsequenz. Aufgrund dieser Eigenschaften werden nun also durch die entsprechenden individuellen HLA-Moleküle nur ganz bestimmte Peptide selektiert und CD8^+^-T-Zellen präsentiert, um von diesen ggf. erkannt zu werden.

Diese Zusammenhänge sind für die Entwicklung neuer antigenspezifischer Krebsimmuntherapien essenziell. So lässt sich etwa davon ableiten, dass sich für viele Mutationen, die auf DNS-Ebene nachgewiesen werden können, keine entsprechenden Peptide auf den HLA-Molekülen von malignen Tumoren finden lassen. Beim malignen Melanom etwa findet sich beispielsweise nur ein minimal kleiner Anteil der genetischen Mutationen als mutiertes Neoantigen auf den HLA-Molekülen des Patienten repräsentiert wieder [19].

Auch für HLA-DR-, HLA-DQ- und HLA-DP-Moleküle (der Klasse II) gelten entsprechende Regeln. Die dort präsentierten Peptide sind zwar etwas weniger strikten Motivregeln unterworfen und HLA-Klasse-II-Moleküle sind etwas weniger polymorph als ihre HLA-Klasse-I-Gegenstücke – etwas über 7000 Allele sind bisher bekannt –, was aber auch in diesem Fall nur zu einer stichprobenartigen Auswahl von (Tumor‑)Antigenen führt. CD4^+^-T-Zellen, die solche HLA-Klasse-II-präsentierten Peptide erkennen können, nehmen ganz zentrale Funktionen des adaptiven Immunsystems wahr.

In der Konsequenz heißt das, dass bei der Entwicklung von Impfungen gegen maligne Tumoren ein undifferenziertes Vorgehen in der Regel ungeeignet ist, weshalb eine an die individuellen Gegebenheiten angepasste Strategie notwendig wird und jeweils geeignete tumorspezifische HLA-Peptide als Zielstrukturen im Einzelfall erst charakterisiert und ausgewählt werden müssen.

### Welche pharmazeutischen Formulierungen, Adjuvanzien und Impfrouten sind effektiv?

Es sind also die T‑Zellen, die gegen Krebszellen wirksam werden können. Was T‑Zellen erkennen, sind dabei stets MHC-restringierte Peptide, wie oben beschrieben. Würde man nun allerdings reine Peptide für eine Impfung gegen maligne Tumoren verwenden, wäre das Vorgehen mit Sicherheit ineffektiv, im schlimmsten Falle sogar kontraproduktiv, weil so T‑Zellen gehemmt werden könnten (Anergie). Entsprechend ist es für solche Impfstoffe unumgänglich, Adjuvanzien genannte Hilfsstoffe zu verwenden, die dazu geeignet sind, eine Immunantwort überhaupt erst zu induzieren und dann zu verstärken.

Immunisierungen mit Peptiden als mögliche Immuntherapie bei Krebs waren bisher nicht effektiv. Zahlreiche Antigenformulierungen und Rezepturen, etwa mit Proteinen, DNS, RNS, aber auch Virus- oder Bakterienkonstrukten, werden noch oder wurden bereits in klinischen Studien getestet, meistens kombiniert mit Adjuvanzien. Eine Übersicht über diese Ansätze findet sich in einem kürzlich publizierten Übersichtsartikel [20]. Was Peptidvakzinierungen betrifft, ist besonders die Verwendung einer Wasser-in-Öl-Emulsion (Letzteres ist auch als inkomplettes Freund-Adjuvans bekannt) als Erfolg versprechend beschrieben worden, insbesondere in Kombination mit Toll-like-Rezeptor(TLR)-Agonisten wie CpG (TLR9; [21]) oder Poly-IC (TLR3; [22]) sowie dem TLR2-Agonisten PAM_3_Cys [23] und davon abgeleiteten Substanzen [24].

Bislang fehlen aber systematische vergleichende Untersuchungen zu unterschiedlichen Impfrouten, Adjuvanzien und Formulierungen von Vakzinen zur Induktion von Immunität gegen maligne Tumoren. Allerdings erscheint ein solcher Ansatz bislang auch nicht zweckmäßig, insbesondere vor dem Hintergrund, dass für viele Ansätze die Induktion von T‑Zellen zwar bereits gezeigt werden konnte und auch anekdotische Berichte über die Effektivität solcher Vakzinierungen existieren, bis heute aber keine effektive Strategie mit ausreichender Evidenz nachgewiesen werden konnte.

Im klinischen Alltag sind therapeutische Impfstoffe gegen Krebs mangels Erfolges bisher nicht etabliert. Im Gegensatz dazu stellen prophylaktische Impfungen gegen Krankheitserreger eine ganz wesentliche Errungenschaft dar und gehören zu den wichtigsten und effektivsten präventiven Maßnahmen der modernen Medizin. Trotz jahrzehntelanger intensiver Forschung an Impfstrategien gegen maligne Tumoren zeichnen sich bisher aber immer noch kaum klinische Erfolge ab und das Feld bleibt daher aktuell zersplittert und sehr komplex. Die seit der Einführung von immuncheckpoint-blockierenden Antikörpern erzielten Erfolge in bestimmten malignen Tumoren unterstreichen allerdings, dass Krebserkrankungen sehr wohl durch das Immunsystem kontrollierbar sind und effektiv bekämpft werden können. Allerdings haben sich alle diese Therapieansätze bisher nur bei wenigen bestimmten Krebserkrankungen als effektiv herausgestellt und sie sind daher längst nicht für alle Patienten Erfolg versprechend.

Da keine der bisher untersuchten Strategien einen klaren Nachweis für ihre Überlegenheit erbringen konnte, kommen grundsätzlich viele verschiedene Ansätze für eine Vakzinierung infrage. Entsprechend können die gewünschten Antigene beispielsweise in Form von DNS oder RNS codiert eingesetzt werden oder aber in ihrer eigentlichen natürlichen Form als Peptide verwendet werden. Weitere Möglichkeiten sind zellbasierte Therapien, etwa mittels viraler Vektoren oder beladener dendritischer Zellen.

## Bisherige klinische Studien

Für viele der genannten therapeutischen Ansätze konnte ein immunologisches Ansprechen in klinischen Studien nachgewiesen werden. Allerdings waren diese Immunantworten in der Regel nicht sehr stark und mussten etwa durch *In-vitro-*Stimulation von T‑Zellen verstärkt werden, um überhaupt messbar zu sein [25]. So zeigten viele publizierte Studien mit Einzelantigenen zwar ein immunologisches Korrelat, blieben aber letztlich ohne relevanten belegbaren klinischen Nutzen [26]. Neben einer Vielzahl von Studien, die mit kleinen Fallzahlen und in frühen klinischen Phasen selbstverständlich keine überzeugenden klinischen Ergebnisse liefern konnten, gibt es einige wenige große randomisierte kontrollierte klinische Studien (englisch „randomized controlled trial“ (RCT)). So lieferte beispielsweise eine Studie mit einem Multipeptidimpfstoff in Kombination mit GM-CSF in der frühen klinischen Entwicklung vielversprechende Immunantworten und erste Hinweise auf ein klinisches Ansprechen [25]. Allerdings konnte die nachfolgende Phase-III-RCT-Studie keine relevante Induktion von vakzinspezifischen T‑Zellen und folglich auch keinerlei klinisch relevante Effekte zeigen [27]. Als ein möglicher Grund für die große Diskrepanz zwischen den Resultaten der frühen klinischen Prüfung und den enttäuschenden Ergebnissen der nachfolgenden klinischen Studien wurde der erst in der Phase-III-Studie zusätzlich verabreichte Tyrosinkinaseinhibitor Sunitinib identifiziert, der im Verlauf der klinischen Entwicklung beim metastasierten Nierenzellkarzinom therapeutischer Standard geworden war. Als mögliche Erklärung für diese Ergebnisse können beispielsweise präklinische Untersuchungen angeführt werden, die nahelegen, dass Tyrosinkinaseinhibitoren die Induktion peptidspezifischer T‑Zellen verhindern können [28]. Letztlich blieben alle uns bekannten Studien in der späten klinischen Entwicklung in den letzten 30 Jahren bezogen auf einen klar belegbaren klinischen Nutzen stets negativ.

### Eigenschaften von Multipeptidvakzinen

Ein generisches Problem, das sich insbesondere im Kontext von Peptidvakzinen stellt, ist die Frage nach der richtigen Auswahl von geeigneten Zielantigenen für eine effektive T‑Zellantwort. Prinzipiell ist es möglich, nur ein einzelnes Antigen für einen Impfstoff auszuwählen, allerdings sprechen triftige Gründe dagegen. Beispielsweise erscheint der Einsatz von multiplen Antigenen schon deshalb sinnvoll, weil neben HLA-Klasse-I- auch HLA-Klasse-II-restringierte Peptide enthalten sein sollten, die eine CD4^+^-T-Zellantwort induzieren können und die für die Entwicklung einer produktiven Immunantwort wesentlich sind. Zudem können kurze Peptide, so wie sie auf HLA-Klasse-I-Molekülen natürlich vorkommen, verwendet werden, aber auch verlängerte Peptide können zum Einsatz kommen, von denen man annimmt, dass sie nach entsprechender Prozessierung zu spezifischen Immunantworten führen. Ein weiterer relevanter Vorteil von synthetischen Peptiden, im Vergleich zu verschiedenen anderen therapeutischen Ansätzen ist, dass Aminosäuresequenzen einen sehr genau definierbaren Wirkstoff darstellen, der grundsätzlich kurzfristig und nach spezifischen Vorgaben herstellbar ist. Andererseits wird dadurch natürlich auch das Spektrum möglicher Immunantworten begrenzt, was wiederum die Auswahl der Zielantigene sehr zentral macht. Ein wesentlicher Aspekt dabei ist die Einschränkung, dass für die Auswahl von HLA-Klasse-I-Liganden der jeweilige persönliche HLA-Genotyp eine wesentliche Rolle spielt. Dies kann durch die individuelle Analyse von Zielstrukturen, beispielsweise durch eine massenspektrometrische Analyse von HLA-gebundenen Peptiden [29, 30], oder aber durch Stratifizierung der Patienten entsprechend ihres HLA-Allotyps adressiert werden. Vakzinierungsstudien mit solchen synthetischen Peptiden in verlängerter Form zeigten bei Frühformen des Vulvakarzinoms eine klinische Wirksamkeit. Allerdings muss hier als Einschränkung beachtet werden, dass in diesem Fall die durch die Impfung induzierte T‑Zellimmunität primär gegen das ursächliche humane Papillomvirus 16 (HPV 16) und nicht gegen die zellulären Tumorantigene gerichtet war [31].

### Individualisierte Vakzine

Zusätzlich wurden aus genomischen Mutationen in malignen Melanomen HLA-bindende Peptide vorhergesagt und diese dann als synthetische Peptidimpfstoffe individualisiert hergestellt und gemeinsam mit dem TLR3-Agonisten Poly-ICLC als Adjuvans verabreicht. Die dabei induzierten Immunantworten beschränkten sich allerdings vor allem auf polyfunktionelle CD4^+^-T-Zellen [32]. Bei lediglich 6 behandelten Melanompatienten, die teilweise zusätzlich noch eine Behandlung mit Immuncheckpoint-Inhibitoren erhielten, muss diese Studie im Ergebnis sicherlich eher anekdotisch bleiben. Ähnliches gilt auch für eine entsprechende Strategie, bei der analog RNS mit solchen mutierten Sequenzen bei 13 Patienten eingesetzt wurde [33]. In beiden Studien konnten starke vakzinspezifische T‑Zellantworten nachgewiesen werden, jedoch konnte nur in 2 Patienten in der letztgenannten Studie auch gezeigt werden, dass die induzierten T‑Zellen tatsächlich die Tumorzellen erkennen, was durch die Infiltration eines entnommenen Tumorstücks mit antigenspezifischen T‑Zellen *ex vivo* belegt werden konnte. Dabei steht natürlich außer Frage, dass die klinische Relevanz noch klarer belegt werden muss und die Anzahl der behandelten Patienten für valide Aussagen bisher noch nicht ausreicht.

Was die angeführten Studien aber zumindest zeigen können, ist, dass sich das Konzept der individuellen Formulierung eines Arzneimittels als Tumorvakzine grundsätzlich bei Tumorerkrankungen umsetzen lässt [34]. Dafür mussten bereits wesentliche Herausforderungen bewältigt werden, beispielsweise mit maschinellem Lernen [32] oder anderen Ansätzen individuelle Zielstrukturen zu definieren, um ein Medikament nach entsprechenden Spezifikationen kurzfristig herzustellen und freigeben zu können. Eine erste klinische Studie in Glioblastompatienten konnte aktuell zeigen, dass die Behandlung von Patienten mit einer solchen Strategie machbar ist [22]. Auch in dieser Studie konnten starke vakzinspezifische T‑Zellantworten belegt werden. Von besonderem Interesse für solche Strategien sind Tumoren mit hoher Mutationslast, in denen sich beispielsweise mutierte Peptide auf HLA nachweisen lassen [19], was in anderen malignen Tumoren bisher aber nur in Ausnahmefällen gelingt [35–37].

Es stellt sich damit weiterhin die Frage nach der optimalen Auswahl der Tumorantigene für Impfstoffe [38]. Auf Basis genomischer Tumormutationen vorhergesagte, aber nicht validierte mutierte Neoantigene sind sicherlich nicht der richtige Weg. Ebenso wichtig und dringend ist die Suche nach geeigneten Adjuvanzien, die eine effektive Immunantwort hervorrufen, um endlich eine klinische Wirksamkeit von Impfstrategien gegen maligne Tumoren zu erreichen.

## Fazit

Um erfolgreiche therapeutische Impfstrategien gegen Krebs zu entwickeln, ist eine detaillierte Kenntnis der immunologischen Vorgänge, aber auch der molekularen Basis des individuellen Tumors notwendig. Dabei werden Erfahrungen zu sammeln sein, wie man in diesen Therapieansätzen Adjuvanzien und weitere Immunmodulatoren sowie etablierte Therapiemodalitäten effektiv kombiniert, um eine klinische Wirksamkeit zu erreichen, und es muss geklärt werden, in welchem Stadium und bei welchen Tumorerkrankungen sich diese Ansätze am besten eignen.

Basierend auf diesen Ausführungen und auf eigenen Vorarbeiten schlagen die Autoren folgendes allgemeines Konzept vor:

Vom Patienten wird eine Tumor- und soweit möglich auch eine Normalgewebsprobe desselben Organs entnommen. Die dort auf HLA-Molekülen präsentierten Peptide werden aus den Proben isoliert und flüssigchromatografisch aufgetrennt und danach massenspektrometrisch charakterisiert. Mit dieser Technologie lassen sich derzeit zumeist ≥5000 HLA-Klasse-I-gebundene Peptide sowie eine große Anzahl von HLA-Klasse-II-Peptiden aus entsprechenden Gewebeproben analysieren [19, 35]. Durch den Vergleich der patientenindividuellen Peptide aus Tumor- bzw. Normalgewebe sowie den Abgleich mit bereits bekannten Peptiden aus verschiedenen Geweben anderer Individuen, die in Datenbanken verfügbar sind (z. B. hla-ligand-atlas.org), können für eine individualisierte Vakzinierung geeignete Peptide bestimmt werden. Erfahrungsgemäß gibt es jeweils Dutzende Peptide, die sich nur auf dem jeweiligen Tumor eines bestimmten Patienten finden lassen, die also tumorspezifisch sind. Von diesen werden dann diejenigen nach verschiedenen zusätzlichen Eigenschaften ausgewählt, etwa nach schon früher etablierten Immunantworten in anderen Patienten, oder Peptide die bereits als Tumorantigene bekannt sind. Gleichzeitig erfolgt die DNS-Sequenzierung des individuellen Tumors, um entsprechende Mutationen zu finden. Kann man durch massenspektrometrische Analyse entsprechende Peptide als HLA-Liganden finden, können diese dann die oben beschriebene Auswahl nichtmutierter Tumorantigene ergänzen.

Aus den so zusammengestellten Peptiden wird eine Selektion von bis zu 10 Peptiden als individualisiertes Arzneimittel hergestellt, mit einem Adjuvans formuliert und zusammen mit einer weiteren Komponente zur Anmischung der Vakzine (Mixing Kit) zur Verabreichung abgegeben. Ein für diesen Zweck vermutlich besonders geeignetes Adjuvans ist das Lipopeptid XS15 [24], das kurzfristig in ersten klinischen Studien getestet werden soll (eine entsprechende Herstellungserlaubnis liegt nun seit dem 24.04.2020 vor). Nach eigenen vorläufigen Ergebnissen kann bereits eine einmalige Vakzinierung mit Peptiden und XS15 emulgiert in Montanide (inkomplettes Freund-Adjuvans) eine starke, direkt *ex vivo *messbare T‑Zellantwort hervorrufen [24], die für mindestens 36 Monate nachweisbar bleibt. Solch eine effiziente Vakzinierung mit Peptiden bzw. mit Sequenzen, die für Peptide codieren, war beim Menschen mit anderen Strategien bisher unbekannt und dies ist auch in der Literatur bislang noch nicht beschrieben worden, soweit den Autoren bekannt. Entsprechend könnte eine therapeutische Vakzinierung mit Peptiden, Montanide und einem effektiven Adjuvans eine vielversprechende Behandlungsoption für Krebspatienten werden.

Wir glauben, dass eine solche individualisierte Vakzinierung bei minimaler Tumorlast oder geringer Resterkrankung vorgenommen werden sollte, im Sinne einer adjuvanten Therapie, etwa kurz nach Tumorresektion. Zusätzlich könnte nach Induktion einer T‑Zellantwort nach Möglichkeit eine zusätzliche Immuncheckpoint-Inhibitortherapie angeschlossen werden, dasselbe gilt, falls ein Patient mit fortgeschrittener Krebserkrankung vakziniert werden sollte.

Aus unserer Sicht hätte eine basierend auf den vorgestellten Überlegungen entwickelte individualisierte Peptidvakzinierung das Potenzial, die onkologische Routinebehandlung für den individuellen Tumorpatienten grundlegend zu verändern.

### Widmung

Wir möchten den hier vorliegenden Artikel dem Andenken an unseren Kollegen und langjährigen Freund Prof. Dr. med. Sebastian P. Haen (1979–2020) widmen.
